# A Retrospective Report on the Infestation and Distribution of Chiggers on an Endemic Rodent Species (*Apodemus latronum*) in Southwest China

**DOI:** 10.3390/vetsci11110547

**Published:** 2024-11-06

**Authors:** Qiao-Yi Liu, Xian-Guo Guo, Rong Fan, Wen-Yu Song, Pei-Ying Peng, Ya-Fei Zhao, Dao-Chao Jin

**Affiliations:** 1 Yunnan Provincial Key Laboratory for Zoonosis Control and Prevention, Institute of Pathogens and Vectors, Dali University, Dali 671000, China; 2 Institute of Microbiology, Qujing Medical College, Qujing 655100, China; 3 Institute of Entomology, Guizhou University, Guiyang 550025, China

**Keywords:** acari, chigger mite, rodent, *Apodemus latronum*, southwest China

## Abstract

Chiggers are the exclusive vector of *Orientia tsutsugamushi*, which is the causative agent of scrub typhus. Based on the field surveys in southwest China between 2001 and 2022, this paper retrospectively reported the chigger infestation and distribution on the large-eared field mouse (*Apodemus latronum*), an endemic rodent species in the region, for the first time. A total of 933 chiggers were collected from 501 mice, and these chiggers were identified as 2 families, 8 genera, and 61 species. The overall infestation prevalence (*P_M_*) and mean abundance (*MA*) of *A. latronum* with chiggers reached 19.76% and 1.86 mites/per mouse, respectively. The chigger infestation indices on adult *A. latronum* (*P_M_* = 38.28%, *MA* = 5.11) were higher than those on juvenile mice (*P_M_* = 12.63%, *MA* = 0.97) with *p* < 0.01, showing an age bias of infestation. The relative fatness (*K*) was used to reflect the nutrition status of the mouse host. The mouse hosts with good nutrition (*K* = 3.4 ± 0.89 g/cm^3^) harbored fewer chiggers than the hosts with poor nutrition (*K* = 2.2 ± 0.90 g/cm^3^) (*p* < 0.01). The infestation indices of chiggers on *A. latronum* obviously fluctuated along different altitude gradients (*p* < 0.01). With the increase in altitudes, the *β* diversity of the chigger community showed a gradually increasing tendency. The spillover chord diagram, which was based on indices of PAC (potential for apparent competition), revealed high spillover potentials of dominant chigger species dispersing from high altitude gradients to the lowest one. The chigger abundance was positively correlated with the mean monthly temperature (tmp), mean monthly humidity (hum), the mean monthly precipitation (pre), and the human footprint (hfp), and it was negatively correlated with the altitude (ele) (*p* < 0.05). The temperature and humidity are the most important factors which influence the chigger infestation.

## Simple Summary

Chiggers are the exclusive vector of scrub typhus. A total of 61 chigger species were identified from *Apodemus latronum*, which is an endemic mouse species in southwest China. There was an age bias; adult *A. latronum* had higher infestation indices of chiggers (*P_M_* = 38.28%, *MA* = 5.11) than juvenile mice (*P_M_* = 12.63%, *MA* = 0.97). The mouse hosts with poor nutrition had higher chigger infestation indices than the hosts with good nutrition. The chigger infestation obviously fluctuated along different altitude gradients, and the *β* diversity of the chigger community showed a gradually increasing tendency from the low to high altitudes. The dominant chigger species had high spillover potentials, dispersing from high to low altitudes. The temperature and humidity are the most important factors influencing the chigger infestation.

## 1. Introduction

Chiggers are a group of tiny arthropods, with more than 3000 species recorded in the world and over 500 species documented in China, are the exclusive transmitting vector of *Orientia tsutsugamushi* (Ot), which is the causative agent of scrub typhus (tsutsugamushi disease) [1,2]. In addition to transmitting Ot, chiggers can be the potential vector of *hantavirus* (HV), which is the pathogen of hemorrhagic fever with renal syndrome, HFRS [3,4]. Scrub typhus and HFRS are zoonotic diseases, and rodents (rats, mice, voles, etc.) are the main infectious source and reservoir hosts of scrub typhus, HFRS, and some other zoonotic diseases [5,6]. Chiggers are ectoparasites on the body surface of other animals (vertebrates, invertebrates, and even some arthropods), and rodents are the main hosts of chiggers [7,8]. During the food intake of chiggers, Ot and *hantavirus* can be transmitted among different rodent hosts and even from rodent hosts to humans [1,9,10]. Scrub typhus and HFRS are widely prevalent in China, and southwest China is an important focus of the diseases [11,12,13].

The large-eared field mouse or Sichuan field mouse (*Apodemus latronum* Thomas) is an endemic rodent species in southwest China, being mainly distributed in some cold and dry areas with high altitudes in the region, e.g., northwestern Yunnan, western Sichuan, and southeastern Tibet [14,15]. In the distribution areas, *A. latronum* is not only a pest in agriculture and forestry but also a reservoir host of scrub typhus, HFRS and other zoonotic diseases [16,17,18]. Although the research on *A. latronum* has involved a few aspects such as the sequencing of the mitochondrial genome, chromosomal karyotypes, cranial measurements and feeding behaviors [17,19,20,21], few studies are about chiggers and other ectoparasites on this endemic mouse species. A previous study revealed that chigger infestations on two sibling mouse species (*Apodemus draco* and *A. ilex*) were quite different, including different species composition, overall infestation, community parameters and dominant chigger species [22], and this leads us to speculate that different rodent species in the same genus may have different susceptibility to chiggers and other ectoparasites due to differences in their behaviors, biological characteristics and geographical distribution. Being an endemic mouse species in southwest China, *A. latronum* may be quite different from other *Apodemus* species in terms of chigger infestation and distribution. Based on the field surveys and taxonomic identification conducted in southwest China from 2001 to 2022, the present paper retrospectively reported the infestation and distribution of chiggers on *A. latronum* for the first time, which is an attempt to learn more about this endemic rodent species in southwest China and its ectoparasites, and then to provide more scientific information for other related in-depth studies in the future.

## 2. Materials and Methods

### 2.1. Collection and Identification of Rodent Hosts and Their Ectoparasitic Chiggers

The original data came from the field surveys and the taxonomic identification of chiggers conducted in southwest China from 2001 to 2022. Southwest China (21°08′–33°41′ N, 97°21′–110°11′ E) is a vast territory with diverse topography, different geographical landscapes, and different types of vegetation and climates, which includes five provincial regions, Yunnan, Sichuan, Guizhou, Chongqing and Tibet (Xizang Autonomous Region) [15,23]. The field surveys were carried out in 114 survey sites within the five provincial regions of southwest China between 2001 and 2022. In field surveys, mousetraps (18 × 12 × 9 cm; Guixi Mousetrap Apparatus Factory, Guixi, Jiangxi, China) baited with fresh peanuts, corn, or other baits were placed at each survey site in the afternoon or evening for capturing rodents (rats, mice, voles, etc.) and other sympatric small mammal hosts (shrews, tree shrews, etc.). In the following morning, the trapped animal hosts were collected with white cloth bags and transported to the field temporary laboratory [24,25]. In the temporary laboratory, each animal host captured was separately placed in a large white plate or a special “double square plate device” to collect its ectoparasitic chiggers after being anesthetized with ether [25,26,27]. The “double square plate device” is recommended for the collection of ectoparasites, and it consists of a white square plate of suitable size placed within a large white square plate. An adequate amount of water is added into the large square plate. This special device is designed to prevent the escape of some ectoparasites (e.g., fleas) that have not been fully anesthetized by ether or those have revived after anesthesia during the collection process. Chiggers are very tiny, and it is very difficult to identify them with the naked eye. In order to collect as many chiggers as possible and to ensure that the numbers of chiggers collected from every animal host are comparable, the thin and tender skin sites (the auricle, outer opening of external auditory canal, groin, and perianal area) where chiggers frequently attach were chosen as fixed collection sites. A lancet or curette (ear scraper) was used to scrape chiggers and chigger-like debris (suspected chiggers) from the skin of each animal host under the help of a magnifier. The collected chiggers were placed into a lidded centrifuge tube containing 70% ethanol for fixation [28,29]. After the collection of chiggers, each animal host was identified into species according to its morphological characteristics, including a series of measurements such body weight, body length, tail length, ear length, and foot length [15,30]. In the laboratory, the chiggers preserved in 70% ethanol were transferred into distilled water to rinse 2–3 times, and the chiggers were separated from other non-chigger debris under a stereomicroscope (Beijing Electronic Optical Equipment Factory, Beijing, China) [25,31,32,33]. The separated chiggers were then mounted on glass slides with Hoyer’s solution. After dehydration, drying, and a transparent process, each glass slide specimen of chigger was carefully observed and measured one by one under a microscope (Olympus Corporation, Tokyo, Japan) for species identification [28,29,34]. After the identification of all the chiggers, the mouse *A. latronum* and its chiggers were selected as the subject of the present study.

**Figure 1:**
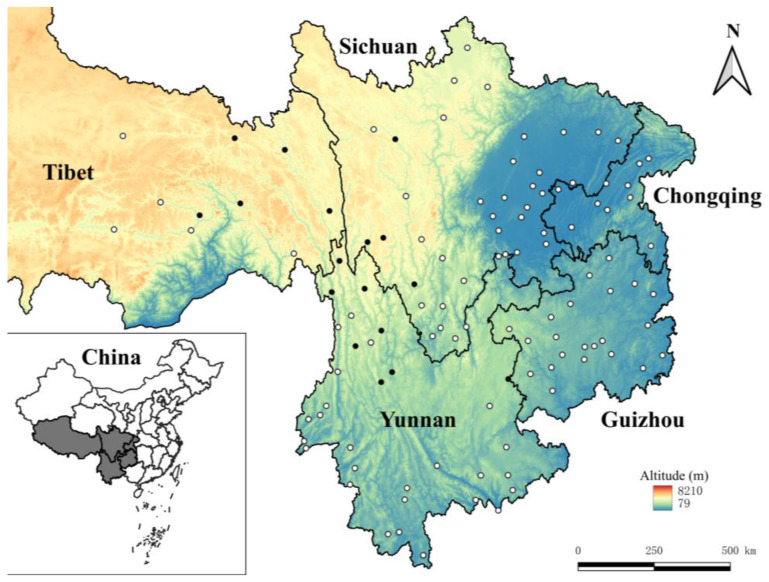
Total 114 survey sites and the 17 sites where *Apodemus latronum* were found in southwestern China (2001–2022). Annotation: Black dots are the 17 sites where *Apodemus latronum* were captured, and white dots are the sites where no *A. latronum* was captured.

**Figure 2:**
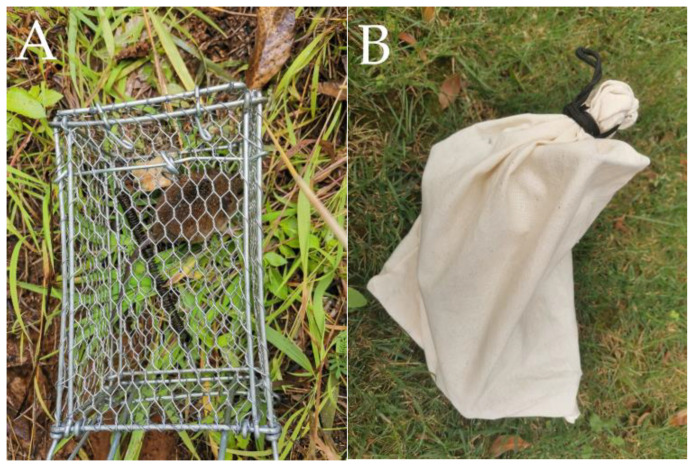
The capture of rodent hosts. Annotation: (**A**) The rodent host was captured with a mousetrap. (**B**) The captured rodent host was collected with a white cloth bag.

**Figure 3:**
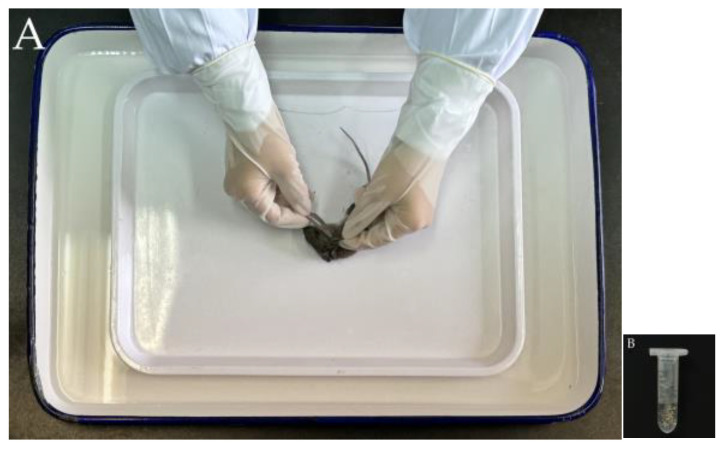
The collection and fixation (preservation) of chiggers. Annotation: (**A**) A lancet was used to scrape chiggers and chigger-like debris from the skin of each animal host in a special “double square plate device”. (**B**) The collected chiggers and chigger-like debris were placed into a lidded centrifuge tube containing 70% ethanol for fixation.

Figure 1 in the Section 2 lists the total 114 survey sites and the 17 sites where *Apodemus latronum* was found in southwestern China (2001–2022), and Figure 2 provides two photos associated with capturing rodent hosts. Figure 3 shows the special “double square plate device” for the collection of chiggers and other ectoparasites as well as the lidded centrifuge tube containing 70% ethanol for the fixation (preservation) of the parasites.

The capture and use of animals (including animal euthanasia) for the research was officially approved by local authorities for wildlife affairs and the Animals’ Ethics Committee of Dali University. The representative specimens were deposited in the specimen repository of the Institute of Pathogens and Vectors, Dali University.

### 2.2. Statistic Analysis

The constituent ratio (*C_r_*, %) was calculated to reflect the percentage of a certain chigger species within the chigger community. The prevalence (*P_M_*, %) was used to calculate the infestation frequency of chiggers on the mice, *A. latronum*. The mean abundance (*MA*, chiggers/per examined host) was used to calculate the average infestation intensity of chiggers on the examined mouse hosts. The mean intensity (*MI*, chiggers/per infested host) was used to calculate the average infestation intensity of chiggers on the infested mouse hosts. These four indices were conventionally used to reflect the infestation status of rodent hosts with chiggers and other ectoparasites [33,35,36]. Four commonly used community indices were calculated to reflect the chigger community on *A. latronum*: the species richness index (*S*), Shannon–Wiener’s diversity index (*H*′), Simpson’s dominance index (*D*), and Pielou’s evenness (*E*) [33,37,38]. The *β*-diversity (Cody index) was used to reflect the diversity difference between any two adjacent sites along different environmental gradients [39,40]. The significance tests for *P_M_* were performed using the chi-square test, and the Wilcoxon test or Kruskal–Wallis test was used for the significance test of *MA* and *MI*. The four community indices such as *S*, *H*′, *D* and *E* were conducted in the “vegan” package of R software (4.3.3 vision) [41]. The formulae were as follows.

(1) $$C_{r}=\frac{N_{i}}{N}\times 100\%$$

(2) $$P_{M}=\frac{H_{i}}{H}\times 100\%\;$$

(3) $$MA=\frac{N_{i}}{H}$$

(4) $$MI=\frac{N_{i}}{H_{i}}\;$$

(5) $$H\prime =-\sum_{i=1}^{S}P_{i}\ln P_{i}$$

(6) $$\;D=1-\sum_{i=1}^{S}P_{i}^{2}$$

(7) $$E=\frac{H\prime }{\ln S}$$

(8) $$\beta =\frac{g\left(h\right)+l(h)}{2}\;$$

In the above formulae, *N_i_* = the number of a certain chigger species (species i), *N* = the total number of all the chigger species; *H_i_* = the number of hosts infested by chiggers, *H* = the total number of hosts examined, *S* = the number of species in the chigger community, *P_i_* = *N_i_*/*N*, *g*(*h*) = the number of species newly emerging at elevation gradient *h*, *l*(*h*) = the number of species decreasing at gradient *h*.

### 2.3. Calculation of Host Relative Fatness

In order to investigate the influence of the nutritional status of *A. latronum* on chigger infestation and community indices, the host relative fatness (*K*) was introduced to reflect the nutritional status of the host, *A. latronum*. The relative fatness (*K*) is a commonly used index to evaluate the nutritional status of rodents and other animals [42,43]. The higher the relative fatness is, the better the nutritional status would be and vice versa. In order to calculate the relative fatness, the body weight and length of each host must be measured beforehand. In the temporary laboratory of each survey site, the body weight and length of each mouse host were precisely measured by an electronic scale and a standard ruler, respectively. The calculation formula of the relative fatness was as follows [44,45].

(9) $$K=\frac{100W}{L^{3}}$$

In the above formula, *K* = the host relative fatness (g/cm^3^); *W* = body weight of host (g); and *L* = body length of host (cm).

### 2.4. Measurement of Species Spillover

The index of PAC (potential for apparent competition) was used to measure the spillover potentials of dominant chigger species on *A. latronum* among different altitude gradients. The PAC index is usually used to study host–parasite interactions [46,47], and it can be also used to measure disperse potentials (spillover potentials) of parasite species among different altitude patches [48,49]. The values of PAC index range from 0 to 1. The higher the index is, the higher the spillover potential would be. The calculation of the PAC index was conducted with the “bipartite” package in R software (Version 4.3.3) [50].

### 2.5. Analysis on Risk Factors Influencing Chigger Abundance

The random forest model, a widely used model in ecological research and practice [51], was used to analyze the potential risk factors which influence the chigger abundance on the surface of the mice, *A. latronum* [52,53]. In the analysis, the number of chiggers (chigger abundance) at each survey site is the dependent variable. The independent variables include the mean monthly temperature (tmp), mean monthly precipitation (pre), monthly average relative humidity (hum), enhanced vegetation index (evi), human footprint (hfp), and altitude (elevation, ele) at each survey site where the mice were collected. Of these potential risk factors, the human footprint (hfp) is an index to reflect the impact of human activities on the natural environment [54,55]. The enhanced vegetation index (evi) is a calculated index that indirectly reflects the vegetation coverage [56,57]. The dimensional units of the six independent variables, however, are not uniform. For example, the dimensional units of the temperature (tmp) are degrees Celsius (°C), and the dimensional units of the altitudes (ele) are meters (m). Prior to random forest analysis, the six independent variables (tmp, pre, hum, evi, hfp, ele) were standardized by z-score standardization to eliminate the effect of variable unit inconsistencies. The data of tmp, pre and hum were obtained from the National Earth System Science Data Center (https://www.geodata.cn/main/, accessed on 23 September 2023), while the ele data came from our survey records. The human footprint data were obtained from the open access figure share website (https://figshare.com/, accessed on 14 July 2024) [58], and those of evi were obtained from the Earthdata website (https://www.earthdata.nasa.gov, accessed on 14 July 2024). The z-score standardization was conducted with the “zscorer” package in R software (Version 4.3.3) [59]. The random forest analysis was conducted with the “rfPermute” [60] and “psych” packages [61], and the result was visualized with “ggplot2” in R software (Version 4.3.3) [62].

## 3. Results

### 3.1. Species Diversity and Infestation of Chiggers on A. latronum

**Figure 4:**
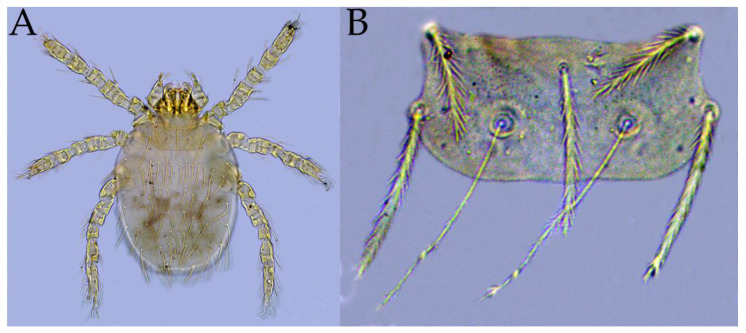
Two micrographs of the dominant and vector chigger species, *Leptotrombidium rupestre*. Annotation: (**A**) The whole mite of *L. rupestre* (×400). (**B**) The scutum of *L. rupestre* (×1000).

Among the 114 survey sites in southwest China, *A. latronum* (mouse host) was found in 17 sites (Figure 1). As mentioned in the Section 2, *A. latronum* and other rodent hosts were captured with mousetraps, and chiggers were conventionally collected and preserved (fixed) in 70% ethanol (Figure 2 and Figure 3). A total of 998 chiggers were collected from the body surface of 501 *A. latronum* mice. Of the 998 collected chiggers, 933 chiggers were identified as 2 families, 8 genera and 61 species, and 65 chiggers remained unidentified because of damaged specimens, obscured structures or suspected new species. The unidentified 65 chiggers were not included in the statistics of this study. The overall infestation prevalence (*P_M_*), mean abundance (*MA*) and mean intensity (*MI*) of chiggers on *A. latronum* were as follows: *P_M_* = 19.76% (99/501), *MA* = 1.86 chiggers/per examined mouse (933/501), and *MI* = 9.42 chiggers/per infested mouse (933/99). Of the 61 identified chigger species, four were dominant species, and they are *Leptotrombidium bayanense*, *Neotrombicula tongtianhensis*, *Leptotrombidium rupestre*, and *Leptotrombidium yongshengense*. The total constituent ratio (*C_r_*) of the four dominant chigger species reached 50.17% of the total 61 species (Table 1). The total *C_r_* value of the remaining 57 chigger species is below 50%, with the *C_r_* of each species being less than 5%. Of the four dominant chigger species, *L. rupestre* is an important potential vector of scrub typhus [63,64], and Figure 4 lists two photos of this dominant and vector species, which were photographed under a microscope.

### 3.2. Variations of Infestation and Community Indices of Chiggers Along Different Altitude Gradients

**Figure 5:**
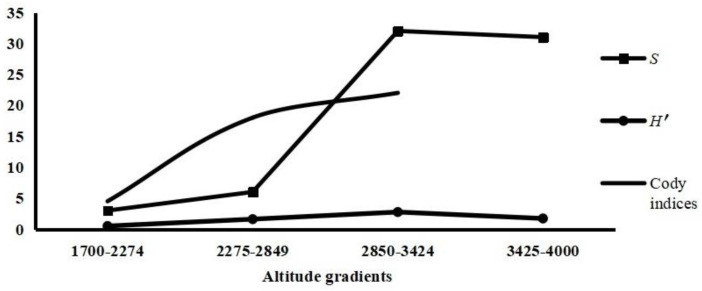
The fluctuation of species richness (*S*), diversity index (*H*′) and Cody index of chigger community on *Apodemus latronum* along different altitude gradients in southwest China (2001–2022).

The captured 501 *A. latronum* were only distributed within the altitude range of 1700–3990 m. According to the original records obtained from the field surveys, the altitude range of 1700–3990 was divided into four altitude gradient (1700–2274 m, 2275–2849 m, 2850–3424 m, and 3425–4000 m) to compare the variations in the community indices of the chigger community along different gradients. The community indices (*S*, *H*′, *D*, *E*, and Cody index) of chiggers were calculated separately for each altitude gradient. Of the four altitude gradients, the infestation indices of chiggers on *A. latronum* in the altitude 1700–2274 m (*P_M_* = 33.33%, *MA* = 4.83, *MI* = 14.50) were the highest, and those in the altitude 2275–2849 m (*P_M_* = 1.61%, *MA* = 0.08, *MI* = 5.00) were the lowest with *p* < 0.01. With the increase in altitudes, the *β* diversity (Cody index) of the chigger community showed a gradually increasing tendency (Table 2, Figure 5).

### 3.3. Variations of Infestation and Community Indices of Chiggers with Different Host Statuses

The results showed that the infestation and community indices of chiggers varied with different host statuses, including different sexes, ages and relative fatness. The number of chigger species (55 species) on the male mice (*A. latronum*) was much higher than that (26 species) on the female ones. The infestation prevalence (*P_M_* = 20.25%), mean abundance (*MA* = 2.31) and mean intensity (*MI* = 11.42) of chiggers on the male mice were higher than the infestation indices on the female mice (*P_M_* = 18.68%, *MA* = 1.11, *MI* = 5.94), but the infestation differences between the males and females were not statistically significant (*p* > 0.05). The infestation indices of chiggers on adult *A. latronum* (*P_M_* = 38.28%, *MA* = 5.11) were much higher than those on juvenile mice (*P_M_* = 12.63%, *MA* = 0.97) with *p* < 0.01, showing an age bias in the infestation (Table 3). Based on the body weight and length of the hosts *A. latronum*, the relative fatness of each mouse was calculated. According to the calculated results of the relative fatness, the mouse hosts were divided into two nutrition groups, the poor nutrition group with the relative fatness *K* = 2.2 ± 0.90 g/cm^3^, and the good nutrition group with *K* = 3.4 ± 0.89 g/cm^3^. The results showed that the mouse hosts in the good nutrition group had lower chigger infestation (*P_M_* = 13.68%, *MA* = 0.99) than the hosts in the poor nutrition group (*P_M_* = 37.8%, *MA* = 5.13) with *p* < 0.01. The community indices (*S*, *H*′, *E*, *D*) of chiggers also varied with the different status of the mouse host (Table 3).

### 3.4. Species Spillover of Dominant Chiggers

**Figure 6:**
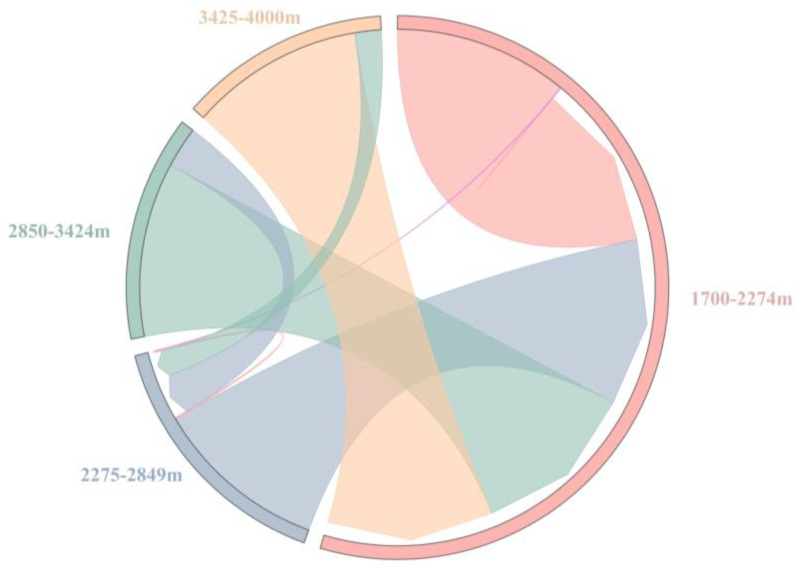
The spillover chord diagram of dominant chigger species on *A. latronum* among different altitude gradients based on PAC indices. Annotation: The arrow size reflets the spillover potentials of dominant chigger species from one altitude gradient to another.

The calculated PAC indices of four dominant chigger species in different altitude gradients were as follows: PAC = 0.23 for 1700–2274 m, PAC = 0.75 for 2275–2849 m, PAC = 0.63 for 2850–3424 m, and PAC = 0.51 for 3425–4000 m. Based on the values of PAC indices, a “spillover chord diagram” was established as in Figure 6, where the arrow size reflets the spillover potentials of dominant chigger species dispersing from one altitude gradient to another. For example, the size of the orange arrow indicates that dominant chigger species had a spillover potential of dispersing from the highest altitude gradient (3425–4000 m) to the lowest one (1700–2274 m) (Figure 6).

### 3.5. Risk Factors Influencing Chigger Abundance

**Figure 7:**
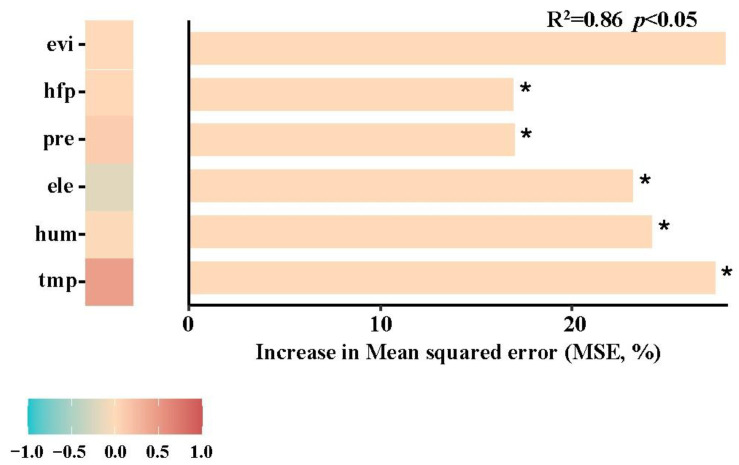
The visualized random forest result for the six potential risk factors influencing chigger abundance. Annotation: tmp = the mean monthly temperature, hum = the mean monthly humidity, pre = the mean monthly precipitation, ele = the altitude, pre = the mean monthly precipitation, hfp = the human footprint, and evi = the enhanced vegetation index. The “*” stands for the statistical significance (*p* < 0.05).

The random forest analysis result was statistically significant (R^2^ = 0.86, *p* < 0.05), and it is visualized in Figure 7. In the coordinate system of Figure 7, the length of each horizontal bar represents the increase ratios of mean square errors (MSEs, %), which reflects the influencing intensity of the corresponding potential risk factors (independent variables) on the chigger abundance (dependent variable). The “*” symbol indicates the statistical significance (*p* < 0.05). The horizontal squares at the bottom of Figure 7 indicate the value changes from the negative correlation (−1 to 0) to the positive correlation (0 to 1). The result revealed that the chigger abundance (dependent variable) was positively correlated with four of the six potential risk factors, the mean monthly temperature (tmp), the mean monthly humidity (hum), the mean monthly precipitation (pre), and the human footprint (hfp) with statistical significance (MSE = 27.47%, 24.17%, 16.99%, and 16.93% respectively, *p* < 0.05). Of these four factors, the “tmp” bar has the longest length, which was followed by the “hum” one. The altitude (ele), however, was negatively correlated with the chigger abundance (MSD = 23.17%, *p* < 0.05). The positive correlation coefficient (MSD = 27.99%) between the enhanced vegetation index (evi) and the chigger abundance was not statistically significant (*p* > 0.05) (Figure 7).

## 4. Discussion

### 4.1. Geographical Distribution of A. latronum

Previous reports showed that there were a few *Apodemus* species in southwest China, including *A. chevrieri*, *A. draco*, *A. ilex*, and *A. agrarius* [22,65,66]. Among these *Apodemus* species, the mouse *A. chevrieri* is one of the dominant rodent species in southwest China with a much larger population and wider geographical distribution than other *Apodemus* species [65]. A total of 1981 *A. chevrieri* mice were once captured from 30 survey sites in southwest China, which is much more than the 501 large-eared field mice (*A. latronum*) in the present study [65]. The result of the present study suggests that *A. latronum* is not the dominant rodent species in southwest China. To date, *A. latronum* has been considered an endemic rodent species in southwest China, being mainly distributed in some cold and dry areas with high altitudes in the region [14,15,23]. In the present study, only 501 *A. latronum* were captured from 17 out of 114 survey sites in southwest China with limited geographical distribution (Figure 1). The 17 sites with *A. latronum* captured were mainly concentrated in the northwestern Yunnan, western Sichuan and southeastern Tibet (Figure 1), where are the cold and dry mountainous areas with high altitudes [67,68,69]. The result further supports the fact that *A. latronum* is an endemic rodent species in southwest China with limited geographical distribution.

### 4.2. Infestation of Chiggers on A. latronum and Medical Significance

Before the present study, few researchers studied the chiggers and other ectoparasites on *A. latronum*; the present paper studied the chigger infestation and distribution on this endemic rodent species in southwest China for the first time. Although few reports focused on chiggers on *A. latronum*, some previous reports did involve chigger species composition and infestation on some other mouse species in the genus *Apodemus* (e.g., *A. chevrieri*, *A. draco* and *A. ilex*), the same genus with *A. latronum*, in southwest China [22,65,66]. The previous reports showed that a total of 107 chigger species were collected from *A. chevrieri* in southwest China with the overall infestation indices *P_M_* = 31.95%, *MA* = 6.32 and *MI* = 19.77. The dominant chigger species on *A. chevrieri* were *Leptotrombidium scutellare* (Nagayo et al.), *L. densipunctatum* Yu et al., and *L. cricethrionis* Wen, Sun and Sun [65]. In contrast, only 14 chigger species were found on the mouse *A. agrarius* in southwest China with low overall infestation indices (*P_M_* = 3.36%, *MA* = 0.36, *MI* = 10.63) and very different dominant chigger species, *L. sialkotense* Vercammen-Grandjean and Langston (*L. jishoum*), *L. rupestre*, and *Schoengastiella novoconfuciana*, Wang et Song [66]. The chigger infestation on two sibling species in the genus *Apodemus*, *A. draco* and *A. ilex*, was previously compared in southwest China. A total of 36 chigger species were identified from *A. draco* with *P_M_* = 10.23%, *MA* = 0.44 and *MI* = 4.26, and the dominant chigger species were *Trombiculindus yunnanus* Wang et Yu, *L. scutellare* and *L. sinicum* Yu et al. And 11 chigger species were identified from *A. ilex* with *P_M_* = 7.14%, *MA* = 0.28 and *MI* = 3.91, and the dominant chigger species were *L. rusticum* Yu, Yang et Gong, *L. densipunctatum*, and *L. gongshanense* Yu et al., which were very different from those on the sibling mouse *A. draco* [22]. The results of the present study revealed that the species composition, species diversity and infestation of chiggers on *A. latronum* in southwest China were also different from those on the above four mouse species in the same genus *Apodemus*. The species diversity (61 species) and infestation indices (*P_M_* = 19.76%, *MA* = 1.86, *MI* = 9.42) on *A. latronum* are much lower than those on *A. chevrieri* but higher than those on *A. draco*, *A. ilex* and *A. agrarius* [22,65,66]. The results indicate that different rodent species in the same genus have different susceptibility to chigger infestation with heterogeneity, which may be mainly associated with the different biological characteristics of different rodent species [70,71]. In addition, *A. latronum* has a limited geographic distribution range, which was mainly in the high-altitude cold areas in western Sichuan, northwestern Yunnan and southeastern Tibet, and this may also influence the different chigger species diversity and infestation on this endemic rodent species. The biological and geographical disparities of different host species often result in the differences of chigger species diversity and infestation [72,73]. The result of the present study further supports the hypothesis that different rodent species in the same genus have different susceptibility to chiggers and other ectoparasites due to differences in their behaviors, biological characteristics and geographical distribution.

In comparison with the juvenile *A. latronum*, the adult mice harbored more chiggers with higher infestation indices (*P_M_* and *MA*, *p* < 0.01). The result indicates the age bias of *A. latronum* when infested with chiggers, which is consistent with previous reports [74]. In the present study, we introduced the relative fatness (*K*) to reflect the nutrition status of the mice. The relative fatness is a common index for evaluating the nutrition status of animals. A high value of relative fatness indicates a good nutrition status and vice versa [42,43]. The result showed that the mouse hosts with poor nutrition (low relative fatness) harbored more chiggers with higher infestation indices than the hosts with good nutrition (Table 3). The above results reflect the influences of host status (age and nutrition status) on chigger infestation. Adult rodents are usually more active and have a wider activity space than juvenile ones in terms of finding food and shelter, as well as mating activities, and this may give the adult rodents more exposure to ectoparasites [35,75,76]. In comparison with the mouse hosts with good nutrition, the hosts with poor nutrition may have lower resistance against parasite invasion, and therefore they would be more likely to be infested with chiggers and other ectoparasites [77,78].

Although there are over 3000 chigger species in the world, not all chigger species can be the effective vector of *O. tsutsugamushi* (Ot), which is the pathogen of scrub typhus. The most effective vector species are mainly those chiggers in the genus *Leptotrombidium* [1,63]. In the present study, a total of 61 chigger species were identified from *A. latronum*, and the dominant chigger species were *L. bayanense*, *Neotrombicula tongtianhensis*, *L. rupestre*, and *L. yongshengense*. Of the four dominant chigger species, three (*L. bayanense*, *L. rupestre* and *L. yongshengense*) belong to the genus *Leptotrombidium*, and *L. rupestre* is an important potential vector of Ot [1,63,64]. *Apodemus latronum* is an important reservoir host and infectious source of scrub typhus [18]. The occurrence of these dominant chigger species of *Leptotrombidium* on *A. latronum* in southwest China may be a potential risk for the transmission of Ot and the focus persistence of scrub typhus in the region. *Neotrombicula tongtianhensis*, however, does not belong to the genus *Leptotrombidium*, and there has been no evidence that *N. tongtianhensis* can serve as the vector or potential vector of scrub typhus. Therefore, the potential risk of *N. tongtianhensis* in the transmission and focus persistence of scrub typhus cannot be determined, and more research is needed in the future.

### 4.3. Influence Factors on Chigger Infestation

In this paper, the PAC index was used to measure the disperse potentials (spillover potentials) of dominant chigger species among different altitude gradients [48,49]. The spillover chord diagram, which was based on PAC indices, revealed the high spillover potentials of dominant chigger species dispersing from one altitude gradient to another.

In the present study, the random forest, a widely used model in ecological research and practice [51,52], was introduced to analyze the influence of six potential risk factors on chigger infestation. The result showed that the chigger abundance was positively correlated with the mean monthly temperature (tmp), mean monthly humidity (hum), mean monthly precipitation (pre), and human footprint (hfp), and it was negatively correlated with the altitude (ele) (*p* < 0.05). The positive correlation between the enhanced vegetation index (evi) and the chigger abundance was not statistically significant (*p* > 0.05). Of the four positive factors (tmp, hum, pre, and hfp), the tmp and hum are the most important risk factors. The results indicate that the chigger infestation on *A. latronum* can be influenced by a series of potential factors, including temperature, humidity, precipitation, altitude, and human activities. The temperature and humidity are the most important factors which positively influence the chigger infestation. The positive influencing effect of the temperature is more than that of the humidity. The altitude (ele), however, can negatively influence the chigger infestation. In southwest China, the temperature, humidity and precipitation are usually high in areas with low altitudes [79,80]. The results of the present study suggest that the chigger abundance on *A. latronum* would be high in the areas with high temperature, high precipitation and low altitudes. Some previous reports have demonstrated that both temperature and precipitation can influence the distribution of chigger mites, and temperature is the most important factor [81,82]. The present study is consistent with previous reports.

### 4.4. Prospects

On the body surface of rodents and other small mammals, there are several taxonomic groups of ectoparasites such as chiggers, gamasid mites, fleas, sucking lice, and even some ticks, and each group of ectoparasites includes many species [83,84,85,86,87]. Due to the special difficulty of identifying all the groups of ectoparasites, it is challenging to study all ectoparasite groups as a whole. The present study only reported chiggers on *A. latronum* in southwest China, and it did not cover all the groups of ectoparasites on the mouse. In fact, different groups of ectoparasites (chiggers, gamasid mites, fleas, sucking lice, and ticks) often co-exist on the same species of rodents, forming an ectoparasite community as a whole. In future studies, it is still worthwhile and necessary to study different groups of ectoparasites as a whole community in order to reveal the overall picture of the ectoparasite community and further elucidate the ecological relationships among different taxonomic categories of ectoparasites.

## 5. Conclusions

The large-eared field mouse, *Apodemus latronum*, is an endemic rodent species in southwest China with limited geographical distribution. It is common for *A. latronum* to be infested with chiggers, and the infestation fluctuates along different altitude gradients. The adult mice are more susceptible to chigger infestation than the juvenile ones. The poor nutrition of mice would increase the infestation burden of chiggers. The chigger infestation is influenced by a series of potential risk factors, including temperature, humidity, precipitation, altitude, and human activities. The temperature and humidity are the most important factors which positively influence the chigger infestation.

## Figures and Tables

**Table 1:** The infestation of four dominant chigger species on *Apodemus latronum* in southwest China (2001–2022).

Dominant Chigger Species	Constituent Ratio (*C_r_*)%	Prevalence (*P_M_*)%	Mean Abundance (*MA*)	Mean Intensity (*MI*)
*Leptotrombidium bayanense*	29.69	2.40	0.55	23.08
*Neotrombicula tongtianhensis*	8.15	1.00	0.15	15.20
*Leptotrombidium rupestre*	6.86	1.40	0.13	9.14
*Leptotrombidium yongshengense*	5.47	1.00	0.10	10.20

**Table 2:** Variations of community indices of chigger community on *Apodemus latronum* along different altitude gradients in southwest China (2001–2022).

Altitude Gradients (m)		Community Indices of Chigger Community							
	No. Hosts	*P_M_* (%)	*MA*	*MI*	*S*	*H*′	*D*	*E*	Cody Indices
1700–2274	30	33.33	4.83	14.50	3	0.50	0.75	0.45	4.5
2275–2849	100	1.61	0.08	5.00	6	1.61	0.24	0.90	18
2850–3424	200	27.50	2.23	8.09	32	2.76	0.09	0.80	22
3425–4000	171	22.81	2.63	11.51	31	1.72	0.39	0.50	

**Table 3:** Variations of infestation and community indices of chiggers with different statuses of the host *Apodemus latronum* in southwest China (2001–2022).

Different Statuses of the Host *A. latronum*		Community Indices				Infestation Indices		
	No. Hosts	*S*	*H*′	*E*	*D*	*P_M_* (%)	*MA*	*MI*
Female	182	26	2.82	0.86	0.09	18.68	1.11	5.94
Male	316	55	2.62	0.65	0.09	20.25	2.31	11.42
Adult	330	35	2.26	0.63	0.13	38.28 **	5.11 **	13.35
Juvenile	172	19	1.94	0.66	0.17	12.63 **	0.97 **	7.67
Poor nutrition	128	21	2.01	0.66	0.26	37.80 **	5.13 **	13.58
Good nutrition	94	34	2.24	0.64	0.22	13.68 **	0.99 **	7.23

Annotation: The symbol “**” denotes *p* < 0.01 in the test of statistical significance. The hosts without corresponding records in the original data were not included in the statistical analysis.

## Data Availability

The experimental data used to support the findings of this study are contained within this article and are available from the corresponding author upon request.

## References

[1] Elliott I., Pearson I., Dahal P., Thomas N.V., Roberts T., Newton P.N. (2019). Scrub typhus ecology: A systematic review of *Orientia* in vectors and hosts. Parasit Vectors.

[2] Li J.C. (1997). Trombiculid Mites of China Studies on Vector and Pathogen of Tsutsugamushi Disease.

[3] Wu G.H., Jiang Z.K., Wang L., Ding L.Y., Mao C.Q., Ma B.Y. (2013). Accordance and identification of vector chigger mites of tsutsugamushi disease in China. Chin. J. Hyg. Insect. Equip..

[4] Huang Y., Zhao L., Zhang Z., Liu M., Xue Z., Ma D., Sun X., Sun Y., Zhou C., Qin X. (2017). Detection of a Novel Rickettsia From *Leptotrombidium scutellare* Mites (Acari: Trombiculidae) From Shandong of China. J. Med. Entomol..

[5] Ganjeer T., Patyal A., Shakya S., Parkar S.S., Shukla A., Chandrakar C., Naik V. (2021). Rodent borne zoonoses: A brief review. Pharma Innov..

[6] Blasdell K.R., Morand S., Laurance S.G.W., Doggett S.L., Hahs A., Trinh K., Perera D., Firth C. (2022). Rats and the city: Implications of urbanization on zoonoticdisease risk in Southeast Asia. PNAS.

[7] Moniuszko H., Makol J. (2014). Chigger mites (Actinotrichida: Parasitengona, Trombiculidae) of Poland. An updated distribution and hosts. Ann. Parasitol..

[8] Vercammen-Grandjean P.H., Benoit P.L., Van Mol J.J. (1970). Terrestrial snail a new host for trombiculid larvae. Acta Trop..

[9] Devasagayam E., Dayanand D., Kundu D., Kamath M.S., Kirubakaran R., Varghese G.M. (2021). The burden of scrub typhus in India: A systematic review. PLoS Negl. Trop. Dis..

[10] Xu G., Walker D.H., Jupiter D., Melby P.C., Arcari C.M. (2017). A review of the global epidemiology of scrub typhus. PLoS Negl. Trop. Dis..

[11] Xin H., Sun J., Yu J., Huang J., Chen Q., Wang L., Lai S., Clements A.C.A., Hu W., Li Z. (2020). Spatiotemporal and demographic characteristics of scrub typhus in Southwest China, 2006-2017: An analysis of population-based surveillance data. Transbound. Emerg. Dis..

[12] Yue Y., Ren D., Liu X., Wang Y., Liu Q., Li G. (2019). Spatio-temporal patterns of scrub typhus in mainland China, 2006-2017. PLoS Negl. Trop. Dis..

[13] Yue Y.J., Wang Y.J., Li G.C., Li X.Z., Wang J., Liu Q.Y. (2020). Epidemiological characteristics of scrub typhus in high-incidence areas in the mainland of China, 2006–2018. Dis. Surveill..

[14] Yue H., Liu S., Liu Y., Zhang X., Fan Z. (2016). Mitochondrial genome of the Sichuan field mouse (*Apodemus latronum*). Mitochondrial DNA A DNA Mapp Seq. Anal..

[15] Zhang Z.B. (2022). Chinese Encyclopedia of Plant Protection Rodent.

[16] Ge D., Feijó A., Cheng J., Lu L., Liu R., Abramov A.V., Xia L., Wen Z., Zhang W., Shi L. (2019). Evolutionary history of field mice (Murinae: *Apodemus*), with emphasis on morphological variation among species in China and description of a new species. Zool. J. Linn. Soc..

[17] Chen Z.P., Liu R.Q., Li C.Y., Wang Y.X. (1996). Studies on the Chromosomes of three species of wood mice. Zool. Sci..

[18] Luo Y.Y., Liu S.T., He Q.N., Hong R.D., Zhu J.J., Ai Z.Q., Yin J.X. (2023). *Orientia tsutsugamushi* Infection in Wild Small Mammals in Western Yunnan Province, China. Pathogens.

[19] Kaneko Y. (2011). Taxonomic status of *Apodemus semotus* in Taiwan by morphometrical comparison with *A. draco, A. peninsulae* and *A. latronum* in China, Korea and Myanmar. Mamm. Study.

[20] Wang B., Yang X.D. (2007). Seed Predation of *Apodemus latronum* on 18 Plant Species in Northwest Yunnan. Zool. Res..

[21] Motokawa M., Wu Y., Harada M., Shintaku Y., Jiang X.L., Li Y.C. (2018). Karyotypes of field mice of the genus *Apodemus* (Mammalia: Rodentia) from China. Zool. Res..

[22] Guo Y., Guo X.G., Song W.Y., Lv Y., Yin P.W., Jin D.C. (2023). Comparison of Chiggers (Acari: Trombiculidae, Leeuwenhoekiidae) on Two Sibling Mouse Species, *Apodemus draco* and *A. ilex* (Rodentia: Muridae), in Southwest China. Animals.

[23] Fan Z.X., Liu S.Y., Liu Y., Zhang X.Y., Yue B.S. (2011). How Quaternary geologic and climatic events in the southeastern margin of the Tibetan Plateau influence the genetic structure of small mammals: Inferences from phylogeography of two rodents, *Neodon irene* and *Apodemus latronum*. Genetica.

[24] Ji W., Veitch C.R., Craig J.L. (1999). An evaluation of the efficiency of rodent trapping methods the effect of trap arrangement, cover type, and bait. N. Z. J. Ecol..

[25] Liu Q.Y., Fan R., Song W.Y., Peng P.Y., Zhao Y.F., Jin D.C., Guo X.G. (2024). The Distribution and Host-Association of the Vector Chigger Species *Leptotrombidium imphalum* in Southwest China. Insects.

[26] Wixson S.K., Smiler K.L., Kohn D.F., Wixson S.K., White W.J., John Benson G. (1997). Chapter 9—Anesthesia and Analgesia in Rodents. Anesthesia and Analgesia in Laboratory Animals.

[27] Underwood W., Anthony R. (2020). AVMA Guidelines for the Euthanasia of Animals: 2020 Edition.

[28] Li Z.P., Zhou H.F., Yang Q.G. (2006). Medical Acarology.

[29] Gu Y.M., Wang J.S. (1999). Gamasid Mites and Chigger Mites in Guizhou.

[30] Gu Y.M., Wang J.S. (1995). Chinese Rodents.

[31] Ding F., Jiang W.L., Guo X.G., Fan R., Zhao C.F., Zhang Z.W., Mao K.Y., Xiang R. (2021). Infestation and Related Ecology of Chigger Mites on the Asian House Rat (*Rattus tanezumi*) in Yunnan Province, Southwest China. Korean J. Parasitol..

[32] Peng P.Y., Guo X.G., Ren T.G., Song W.Y. (2015). Faunal analysis of chigger mites (Acari: Prostigmata) on small mammals in Yunnan province, southwest China. Parasitol. Res..

[33] Peng P.Y., Guo X.G., Ren T.G., Song W.Y., Dong W.G., Fan R. (2016). Species diversity of ectoparasitic chigger mites (Acari: Prostigmata) on small mammals in Yunnan Province, China. Parasitol. Res..

[34] Vercammen-Grandjean P.H., Langston R.L. (1975). The Chigger Mites of the World (Acarina: Trombiculidae & Leeuwenhoekiidae). III. Leptotrombidium Complex.

[35] Liu Z., Guo X.G., Fan R., Zhao C.F., Mao K.Y., Zhang Z.W., Zhao Y. (2020). Ecological analysis of gamasid mites on the body surface of Norway rats (*Rattus norvegicus*) in Yunnan Province, Southwest China. Biologia.

[36] Margolis L., Esch G., Holmes J., Kuris A., Schad G. (1982). The use of ecological terms in parasitology (report of an ad hoc committee of the American Society of Parasitologists). J. Parasitol..

[37] Simpson E. (1949). Measurement of Diversity. Nature.

[38] Bush A.O., Lafferty K.D., Lotz J.M., Shostak A.W. (1997). Parasitology meets ecology on its own terms: Margolis et al. revisited. J. Parasitol..

[39] Legendre P. (2007). Studying beta diversity: Ecological variation partitioning by multiple regression and canonical analysis. J. Plant Ecol..

[40] Zhou J.X., Guo X.G., Song W.Y., Zhao C.F., Zhang Z.W., Fan R., Chen T., Lv Y., Yin P.W., Jin D.C. (2022). Preliminary study on species diversity and community characteristics of gamasid mites on small mammals in three parallel rivers area of China. Animals.

[41] Oksanen J., Simpson G., Blanchet F., Kindt R., Legendre P., Minchin P., O’Hara R., Solymos P., Stevens M., Szoecs E. Vegan: Comunity Ecology Package (Version 2.6-4). https://github.com/vegandevs/vegan.

[42] Rogers P., Webb G.P. (1980). Estimation of body fat in normal and obese mice. Br. J. Nutr..

[43] Caldwell A.E., Sayer R.D. (2019). Evolutionary considerations on social status, eating behavior, and obesity. Appetite.

[44] Yang Z.X., Long G., Jin X., Guo Y.W., Liu J. (2013). Relative Fatness Variation of *Anourosorex squamipes* of Different Genders, Ages and Seasons. Guizhou Agric. Sci..

[45] Fang J.M., Wang H.M., Yu X.D. (1995). Analysis of relative fatness indices of rodents. Acta Ecol. Sin..

[46] Morris R.J., Lewis O.T., Godfray H.C. (2004). Experimental evidence for apparent competition in a tropical forest food web. Nature.

[47] Morris R.J., Lewis O.T., Godfray H.C. (2005). Apparent competition and insect community structure towards a spatial perspective. Ann. Zool. Fenn..

[48] Cappellari A., Santoiemma G., Sanna F., D’Ascenzo D., Mori N., Lami F., Marini L.J.E.G. (2022). Spatio-temporal dynamics of vectors of *Xylella fastidiosa* subsp. pauca across heterogeneous landscapes. Entomol. Gen..

[49] Nardi D., Marini L. (2021). Role of abandoned grasslands in the conservation of spider communities across heterogeneous mountain landscapes. Agr. Ecosyst. Env..

[50] Dormann C.F., Fruend J., Gruber B., Dormann M.C.F. Package ‘Bipartite’. https://github.com/biometry/bipartite.

[51] Cutler D.R., Edwards T.C., Beard K.H., Cutler A., Hess K.T., Gibson J., Lawler J.J. (2007). Random forests for classification in ecology. Ecology.

[52] Rigatti S.J. (2017). Random forest. J. Insur. Med..

[53] Wang Q., Wang X., Zhou Y., Liu D., Wang H. (2022). The dominant factors and influence of urban characteristics on land surface temperature using random forest algorithm. Sustain. Cities Soc..

[54] Correa Ayram C.A., Mendoza M.E., Etter A., Pérez Salicrup D.R. (2017). Anthropogenic impact on habitat connectivity: A multidimensional human footprint index evaluated in a highly biodiverse landscape of Mexico. Ecol. Indic..

[55] Sanderson E.W., Jaiteh M., Levy M.A., Redford K.H., Wannebo A.V., Woolmer G. (2002). The Human Footprint and the Last of the Wild: The human footprint is a global map of human influence on the land surface, which suggests that human beings are stewards of nature, whether we like it or not. BioScience.

[56] Liu X., Jiang W., Li J., Wang W. (2017). Evaluation of the Vegetation Coverage Resilience in Areas Damaged by the Wenchuan Earthquake Based on MODIS-EVI Data. Sensors.

[57] Yan E., Wang G., Lin H., Xia C., Sun H. (2015). Phenology-based classification of vegetation cover types in Northeast China using MODIS NDVI and EVI time series. Int. J. Remote Sens..

[58] Mu H., Li X., Wen Y., Huang J., Du P., Su W., Miao S., Geng M. (2022). A global record of annual terrestrial Human Footprint dataset from 2000 to 2018. Sci. Data.

[59] Myatt M., Guevarra E. (2019). Zscorer: Child Anthropometry Z-Score Calculator.

[60] Archer E. (2018). rfPermute: Estimate Permutation p-Values for Random Forest Importance Metrics (2013). J. R Package Version.

[61] Revelle W., Revelle M.W. (2015). Package ‘psych’. J Compr. R Arch. Netw..

[62] Wickham H. (2011). ggplot2. Wiley Interdiscip. Rev..

[63] Traub R., Wisseman C.L. (1974). The ecology of chigger-borne rickettsiosis (scrub typhus). J. Med. Entomol..

[64] Fan M.Y., Walker D.H., Yu S.R., Liu Q.H. (1987). Epidemiology and ecology of rickettsial diseases in the People’s Republic of China. Rev. Infect Dis..

[65] Chen Y.L., Guo X.G., Ren T.G., Zhang L., Fan R., Zhao C.F., Zhang Z.W., Mao K.Y., Huang X.B., Qian T.J. (2022). Infestation and distribution of chigger mites on Chevrieri’s field mouse (*Apodemus chevrieri*) in Southwest China. Int. J. Parasitol. Parasites Wildl..

[66] Chen Y.L., Guo X.G., Ren T.G., Zhang L., Fan R., Zhao C.F., Zhang Z.W., Mao K.Y., Huang X.B., Qian T.J. (2021). A Report of Chigger Mites on the Striped Field Mouse, *Apodemus agrarius*, in Southwest China. Korean J. Parasitol..

[67] Fielding E., Isacks B., Barazangi M., Duncan C. (1994). How flat is Tibet?. Geology.

[68] Li W.J., Peng M.C., Higa M., Tanaka N., Matsui T., Tang C.Q., Ou X.K., Zhou R.W., Wang C.Y., Yan H.Z. (2016). Effects of climate change on potential habitats of the cold temperate coniferous forest in Yunnan province, southwestern China. J. Mountain Sci..

[69] Xu S., Cheng B., Huang Z.F., Shen C.Y. (2022). An Investigation on the Thermal Environment of Residential Courtyards in the Cold Area of Western Sichuan Plateau. Buildings.

[70] Chaisiri K., Gill A.C., Stekolnikov A.A., Hinjoy S., McGarry J.W., Darby A.C., Morand S., Makepeace B.L. (2019). Ecological and microbiological diversity of chigger mites, including vectors of scrub typhus, on small mammals across stratified habitats in Thailand. Anim Microbiome.

[71] Alkathiry H., Al-Rofaai A., Ya’cob Z., Cutmore T.S., Mohd-Azami S.N.I., Husin N.A., Lim F.S., Koosakulnirand S., Mahfodz N.H., Ishak S.N. (2022). Habitat and Season Drive Chigger Mite Diversity and Abundance on Small Mammals in Peninsular Malaysia. Pathogens.

[72] Morand S. (2015). (macro-) Evolutionary ecology of parasite diversity: From determinants of parasite species richness to host diversification. Int. J. Parasitol. Parasites Wildl..

[73] Wulandhari S.A., Paladsing Y., Saesim W., Charoennitiwat V., Sonthayanon P., Kumlert R., Morand S., Sumruayphol S., Chaisiri K. (2021). High prevalence and low diversity of chigger infestation in small mammals found in Bangkok Metropolitan parks. Med. Vet. Entomol..

[74] Xiang R., Guo X.G., Ren T.G., Zhao C.F., Fan R., Zhang Z.W., Mao K.Y., Peng P.Y., Huang X.B., Qian T.J. (2021). Infestation and distribution of mites on the Yunnan red-backed vole (*Eothenomys miletus*) in Yunnan Province of southwest China between 2001 and 2015. Biologia.

[75] Kataranovski M., Mirkov I Fau—Belij S., Belij S Fau—Popov A., Popov A Fau—Petrovic Z., Petrovic Z Fau—Gaci Z., Gaci Z Fau—Kataranovski D., Kataranovski D. (2011). Intestinal helminths infection of rats (*Ratus norvegicus*) in the Belgrade area (Serbia): The effect of sex, age and habitat. Parasite J. La Société Française Parasitol..

[76] Poulin R. (1997). Species richness of parasite assemblages: Evolution and patterns. Annu. Rev. Ecol. Evol. S.

[77] Huang Z.C. (2012). Pollen nutrition affects honey bee stress resistance. Terrestrial. Arthropod Rev..

[78] PG G., Parry H. (1948). Discussion on nutrition and resistance to infection. P Roy Soc. Med..

[79] Qing Y., Zhu-Guo M., Liang C. (2015). A Preliminary Analysis of the Relationship between Precipitation Variation Trends and Altitude in China. Atmos. Ocean. Sci. Lett..

[80] Jiang S., Chen X., Smettem K., Wang T. (2021). Climate and land use influences on changing spatiotemporal patterns of mountain vegetation cover in southwest China. Ecol. Indic..

[81] Dirk Van Peenen P.F., Lien J.-C., Santana F.J., Richard S. (1976). Correlation of chigger abundance with temperature at a hyperendemic focus of scrub typhus. J. Parasitol..

[82] Sasa M. (1961). Biology of chiggers. Annu. Rev. Entomol..

[83] Stanko M., Fricova J., Miklisova D., Khokhlova I.S., Krasnov B.R. (2015). Environment-related and host-related factors affecting the occurrence of lice on rodents in Central Europe. Parasitology.

[84] Lareschi M., Krasnov B.R. (2010). Determinants of ectoparasite assemblage structure on rodent hosts from South American marshlands: The effect of host species, locality and season. Med. Vet. Entomol..

[85] Kowalski K., Eichert U., Bogdziewicz M., Rychlik L. (2014). Differentiation of flea communities infesting small mammals across selected habitats of the Baltic coast, central lowlands, and southern mountains of Poland. Parasitol. Res..

[86] Zajac Z., Kulisz J., Wozniak A. (2020). Flea Communities on Small Rodents in Eastern Poland. Insects.

[87] Krawczyk A.I., van Duijvendijk G.L.A., Swart A., Heylen D., Jaarsma R.I., Jacobs F.H.H., Fonville M., Sprong H., Takken W. (2020). Effect of rodent density on tick and tick-borne pathogen populations: Consequences for infectious disease risk. Parasit. Vectors.

